# Blapstin, a Diapause-Specific Peptide-Like Peptide from the Chinese Medicinal Beetle *Blaps rhynchopetera*, Has Antifungal Function

**DOI:** 10.1128/spectrum.03089-22

**Published:** 2023-05-04

**Authors:** La-Mei Zhang, Min Yang, Sheng-Wen Zhou, Hao Zhang, Ying Feng, Lei Shi, Dong-Sheng Li, Qiu-Min Lu, Zhong-He Zhang, Min Zhao

**Affiliations:** a Institute of Highland Forest Science, Chinese Academy of Forestry, Kunming, China; b College of Forestry, Nanjing Forestry University, Nanjing, Jiangsu, China; c Key Laboratory of Breeding and Utilization of Resource Insects, National Forestry and Grassland Administration, Kunming, China; d Key Laboratory of Bioactive Peptides of Yunnan Province, Kunming Institute of Zoology, Kunming, Yunnan, China; e Kunming College of Life Science, University of Chinese Academy of Sciences, Beijing, China; f Sino-African Joint Research Center, Chinese Academy of Science, Wuhan, Hubei, China; Universidade de Brasilia

**Keywords:** diapause-specific peptide-like peptide, DSP, *Blaps rhynchopetera*, blapstin, antifungals, *Candida albicans*, *Trichophyton rubrum*, fungi, microorganism, pathogens

## Abstract

Drug resistance against bacteria and fungi has become common in recent years, and it is urgent to discover novel antimicrobial peptides to manage this problem. Many antimicrobial peptides from insects have been reported to have antifungal activity and are candidate molecules in the treatment of human diseases. In the present study, we characterized an antifungal peptide named blapstin that was isolated from the Chinese medicinal beetle Blaps rhynchopetera used in folk medicine. The complete coding sequence was cloned from the cDNA library prepared from the midgut of *B. rhynchopetera*. It is a 41-amino-acid diapause-specific peptide (DSP)-like peptide stabilized by three disulfide bridges and shows antifungal activity against Candida albicans and Trichophyton rubrum with MICs of 7 μM and 5.3 μM, respectively. The C. albicans and T. rubrum treated with blapstin showed irregular and shrunken cell membranes. In addition, blapstin inhibited the activity of C. albicans biofilm and showed little hemolytic or toxic activity on human cells and it is highly expressed in the fat body, followed by the hemolymph, midgut, muscle, and defensive glands. These results indicate that blapstin may help insects fight against fungi and showed a potential application in the development of antifungal reagents.

**IMPORTANCE** Candida albicans is one of the conditional pathogenic fungi causing severe nosocomial infections. Trichophyton rubrum and other skin fungi are the main pathogens of superficial cutaneous fungal diseases, especially in children and the elderly. At present, antibiotics such as amphotericin B, ketoconazole, and fluconazole are the main drugs for the clinical treatment of C. albicans and T. rubrum infections. However, these drugs have certain acute toxicity. Long-term use can increase kidney damage and other side effects. Therefore, obtaining broad-spectrum antifungal drugs with high efficiency and low toxicity for the treatment of C. albicans and T. rubrum infections is a top priority. Blapstin is an antifungal peptide which shows activity against C. albicans and T. rubrum. The discovery of blapstin provides a novel clue for our understanding of the innate immunity of *Blaps rhynchopetera* and provides a template for designing antifungal drugs.

## INTRODUCTION

In recent years, morbidity and mortality rates due to fungal infections have been on the rise (1). The incidence of fungal infections has increased significantly over the past 20 years, and in some clinically fungal infection cases, the mortality rate of patients with systemic fungal infections has exceeded 90% (2). The use of antifungal drugs plays a pivotal role in the control of fungal infections. However, with the continuous emergence of drug-resistant strains and the increasing degree of drug resistance, research on new antifungal drugs has become a top priority. It was found that antifungal peptides in insects have a good inhibitory and killing effect on human-, animal-, and plant-pathogenic fungi (3).

When insects are infected by microorganisms, effector molecules, such as antimicrobial peptides (AMPs), are produced in their fat bodies and blood cells, which are rapidly released into the hemolymph to resist the invasion of pathogenic microorganisms (4). Since cecropin, an inducible antibacterial peptide, was first identified in the moth Hyalophora cecropia in the 1980s, insect AMPs have been found in more than 300 insect species (https://aps.unmc.edu/AP/) (5), including the antifungal peptide drosomycin extracted from the hemolymph of Drosophila melanogaster (6), thanatin, which was isolated from the hemolymph of Podisus maculiventri (7), and the diapause-specific peptide (DSP) that was isolated from the leaf beetle Gastrophysa atrocyanea (8). According to different structures and functions, insect AMPs can be roughly divided into the following four categories: cecropins (cecropin and moricin) (9), peptides with excess proline (10), peptides with excess glycine (holotricin 3 and tenecin 3) (11, 12), and cysteine-rich peptides (insect defensins) (13). Insect defensins belong to the fourth class, and the six conserved cysteine residues form three disulfide bonds (Cys1-Cys4, Cys2-Cys5, Cys3-Cys6) whose structure constitutes the Csαβ domain (14). It is worth noting that a DSP isolated from the leaf beetle *G. atrocyanea* contains 41 amino acids and 6 cysteine residues, which lead to the formation of three pairs of disulfide bonds; it has a spatial structure similar to that of drosomycin, but its secondary structure βαββ is significantly different from the Csαβ structure of defensins. Therefore, the DSP does not belong to the insect defensins family (8, 15, 16). The peptide of SeDSP from the beet armyworm Spodoptera exigua, which is similar to the DSP from the leaf beetle *G. atrocyanea*, has been identified from Lepidoptera and has antibacterial activity against Bacillus megaterium. In addition, the antifungal peptide Diapausin-1 from the hemolymph of the lepidopteran insect Manduca sexta (tobacco hornworm) has spectral antibacterial activity against yeast specie *Saccharomyces cerevisia*. This group of peptides mentioned above are classified into the diapausin family (17).

Biofilm on a microbial surface is a microbial community wrapped in a self-produced substrate, usually consisting of polysaccharides, DNA, and proteins in the form of an adsorption (18). About 80% of human infections by pathogenic microorganisms are caused by biofilms (19–21). The main antibiofilm mechanisms of AMPs include destroying biofilm attached to the cell membrane surface (22, 23), interfering with the intracellular signal system (24, 25), degradation of biofilm components, including polysaccharides and biofilm matrix (26, 27), inhibition of the alarm system to avoid the microbial response (28, 29), and downregulation of genes responsible for biofilm formation (30, 31). AMPs can interfere in the early stages of biofilm formation to prevent microbial adhesion on the surface (32). At appropriate polypeptide concentration, the AMPs cause membrane permeability on the biofilm by forming a “ring hole” model or a “carpet” model (33, 34). The membrane permeability causes leakage of ions and metabolites, which leads to depolarization of the transmembrane potential and then to impaired membrane function and eventually rupture (34). Therefore, it is urgent to find new effective drugs to treat Candida albicans and its biofilm infections.

In the present study, we identified, for the first time to our knowledge, a DSP-like peptide in Blaps rhynchopetera, an insect used in folk medicine in Yunnan Province, China. This research investigated the structure and characterization of the new antifungal peptide, a 41-residue peptide with 6 cysteines forming three disulfide bonds similar to DSP. It also inhibited the growth of a range of fungi, including C. albicans and Trichophyton rubrum. These findings suggest that blapstin may be a candidate drug for the treatment of human-pathogenic fungi.

## RESULTS

### cDNA cloning and bioinformatics analysis.

**FIG 1:**
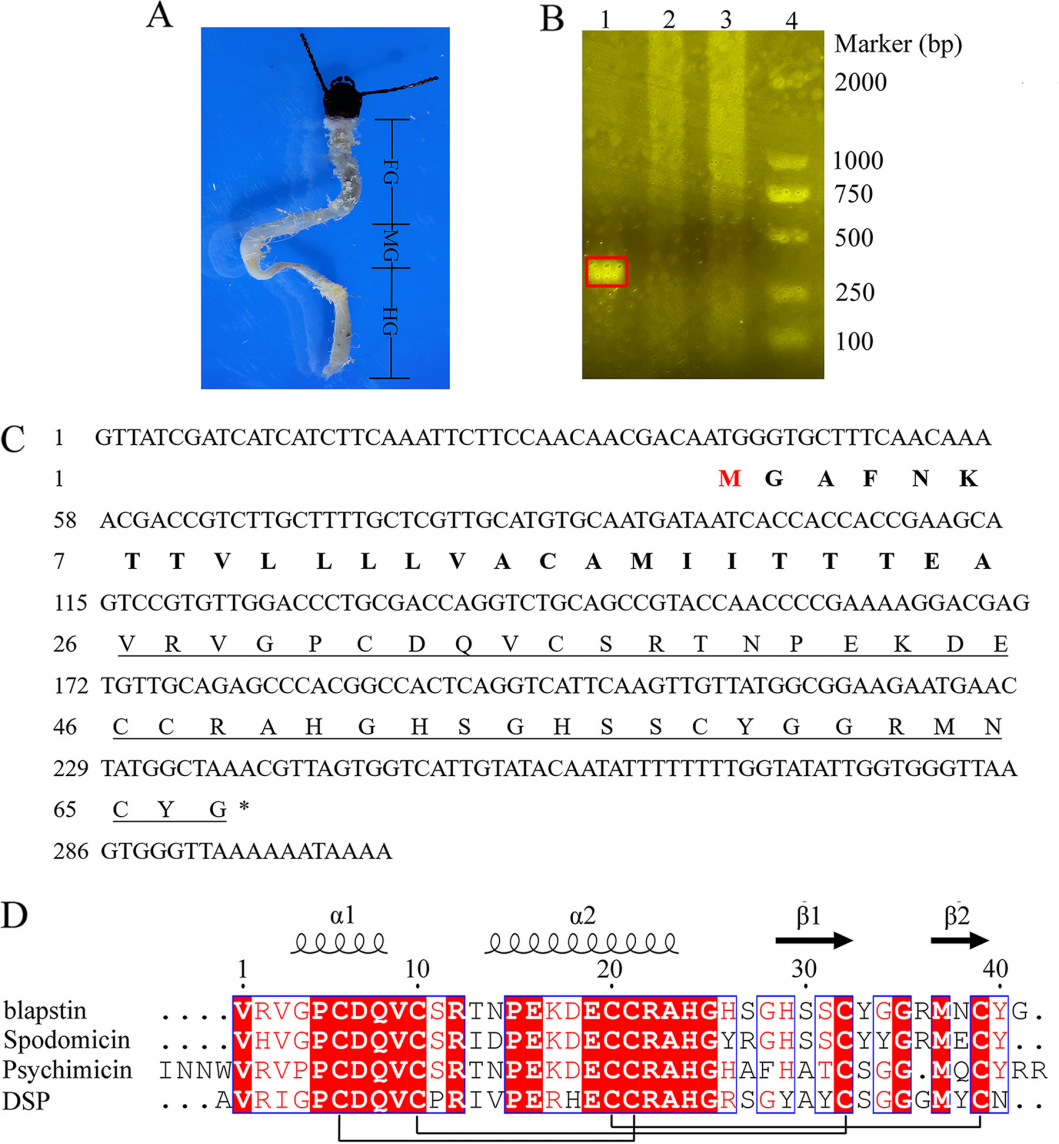
Dissected intestinal tissue of *Blaps rhynchopetera* and the primary structure of blapstin. (A) Intestinal tissue isolated from *B. rhynchopetera*. FG, foregut; MG, midgut; HG; hindgut. (B) PCR products of DNA encoding blapstin were observed by 1% agarose gel electrophoresis; the band was approximately 250 bp. The red box represents the PCR product of DNA encoding blapstin. Lanes: 1, a band appeared indicating that the designed primer pair is correct and the DNA can be sequenced; 2 and 3, a band did not appear, indicating that the designed primer pair was correct and the DNA cannot be sequenced; 4, a band indicating DNA markers. (C) Nucleotide and deduced amino acid sequences of cDNA encoding blapstin. M (marked in red) represents the starting amino acid of blapstin. The mature peptide sequence is underlined. The amino acid sequence in bold represents the signal peptide of blapstin. An asterisk indicates the stop codon. (D) Sequence alignment of blapstin (GenBank accession no. ON754988), spodomicin (GenBank accession no. P83411), psychimicin (GenBank accession no. P83421), and DSP (GenBank accession no. Q8T0W8). The conserved residues are highlighted in red. α1 and α2 indicate α-helixes, and β1 and β2 indicate β-sheets. The disulfide bonds are depicted by connecting lines.

We successfully identified a DSP-like peptide from the transcriptome of the midgut of *B. rhynchopetera* and obtained its full coding sequence from the cDNA library. Total RNA was isolated from the midgut of *B. rhynchopetera* (Fig. 1A). The result indicated that the *A*_260_/*A*_280_ optical density ratio for the total RNA was 2.033, which was well within the optimal range of 1.8 to 2.1. The concentration of mRNA was 236.81 ng/μL, indicating that the isolated total RNA was suitable for cDNA amplification. The cDNA library was successfully constructed using a SMART cDNA library construction kit, and the *A*_260_/*A*_280_ optical density ratio for the double-stranded DNA was 2.005. Finally, the coding sequence of the DSP-like peptide was amplified by PCR using the double-stranded DNA in the cDNA library as a template. The sequencing results indicated that the cloned sequence included a 201-bp open reading frame encoding a 66-amino-acid sequence (Fig. 1B). The predicted complete peptide was MGAFNKTTVLLLLVACAMIITTTEAVRVGPCDQVCSRTNPEKDECCRAHGHSGHSSCYGGRMNCYG with a predicted isoelectric point (pI) of 7.56 and a molecular mass of 7,066.13 Da. The signal peptide was identified using the SignalP 5.0 server. The sequence of the mature peptide without the signal peptide was VRVGPCDQVCSRTNPEKDECCRAHGHSGHSSCYGGRMNCYG. The molecular weight of the mature peptide was predicted to be 4,455.835 Da, and the peptide was named blapstin (GenBank accession no. ON754988) (Fig. 1C). The results of BLAST and FASTA revealed that the predicted protein has high homology (maximum score, >50%) to the diapausin family, including the three peptides spodomicin, psychimicin, and DSP (GenBank accession no. P83411, P83421, and Q8T0W8, respectively) from the Egyptian cotton leafworm (Spodoptera littoralis), the bagworm moth (Oiketicus kirbyi), and the leaf beetle (*G. atrocyanea*), respectively (9, 10) (Fig. 1D).

### Structure of blapstin.

**FIG 2:**
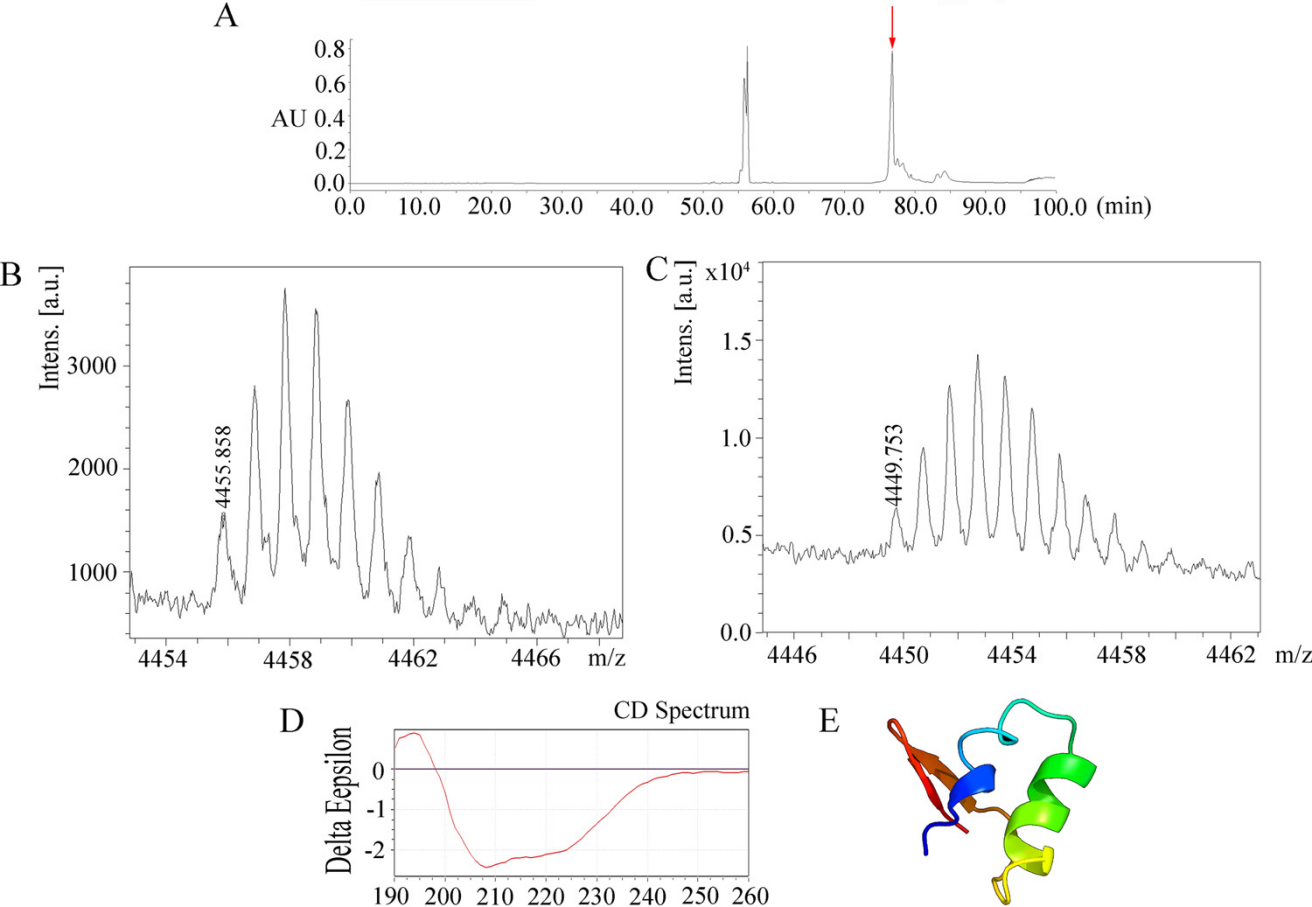
Refolding and secondary structure of blapstin. (A) After refolding, the fractions containing blapstin were eluted by RP-HPLC on a C_18_ column (10 mm by 250 mm; Waters, USA). Each peak was concentrated and identified by MALDI-TOF MS (Autoflex; Bruker Daltonics, USA). The retention time of refolded blapstin was 77 min (red arrow). AU, arbitrary units. (B) The linear peptide molecular weight of blapstin (4,455.858 Da) was identified by mass spectrometry. Intens., intensity; a.u., arbitrary units. (C) The molecular weight of the refolded blapstin peptide (4,449.753 Da) was identified by MS. (D) The secondary structure of blapstin was determined by CD spectrometry; the two-dimensional structure of blapstin was predicted by CD tool software. (E) Predicted three-dimensional structure of blapstin. Different colors represent different secondary structure types: green, α-helix; red, β-sheet; blue, random coil.

To obtain the peptide, the 41-amino-acid blapstin was biochemically synthesized by Sangon Biotech, Ltd. (Shanghai, China), and we then refolded the peptide. Figure 2A shows that the retention time of refolded blapstin was 77 min. The eluted blapstin was collected, lyophilized, and used for further activity analysis. The molecular mass of the purified blapstin was confirmed using matrix-assisted laser desorption ionization–time of flight mass spectrometry (MALDI-TOF MS) (Autoflex; Bruker Daltonics, USA). The measured isotopic molecular mass of the linear peptide was 4,455.853 Da, whereas the isotopic molecular mass of the renatured peptide was 4,449.753 Da [(M + H)^+^, with a mass discrepancy of 10 ppm]. The reduction of 6.043 Da in molecular mass indicates that the three disulfide bonds of blapstin were successfully formed (Fig. 2B and C). Circular dichroism (CD) spectrometry showed that the secondary structure of blapstin was characterized mainly by random curl (34.80%), β-turns (22.54%), and β-sheets (20.66%), in addition to α-helix structures (5.22%) (Fig. 2D), which was consistent with the spatial structure predicted by the PHYRE2 online program (Fig. 2E).

### Expression of blapstin in different tissues of *B. rhynchopetera*.

**FIG 3:**
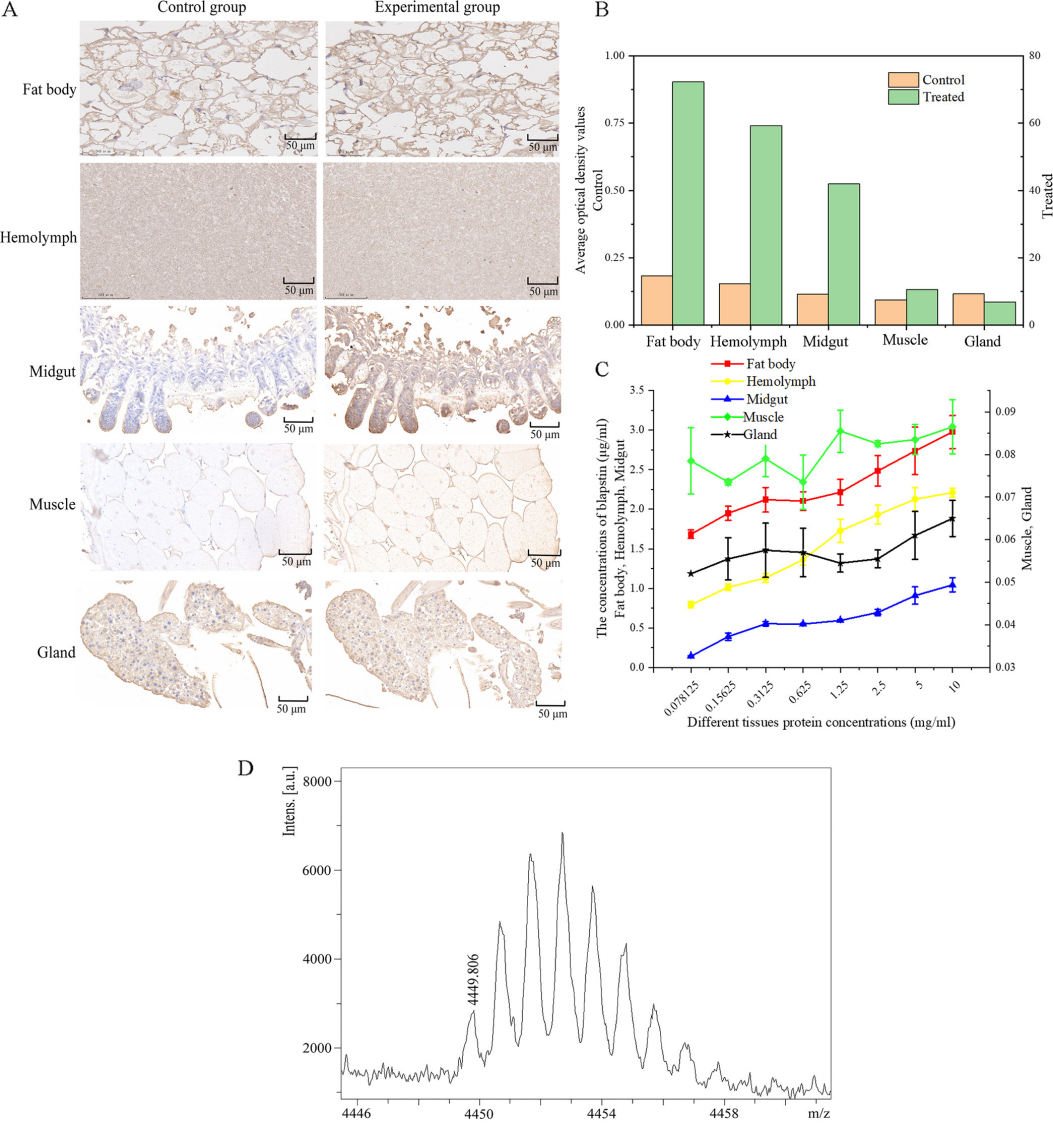
Expression of blapstin in different tissues of *B. rhynchopetera*. (A) Expression of blapstin in different tissues was observed by IHC. The control group consists of tissues stained with IgG from unimmunized rabbit serum. The experimental group includes tissues stained with IgG from immunized rabbit serum. (B) Image J analysis of IHC expression levels in different tissues of *B. rhynchopetera.* (C) ELISA showing the concentrations of blapstin in different tissues. (D) Results of MALDI-TOF MS indicating the presence of blapstin in the midgut of *B. rhynchopetera* with a molecular weight of 4,449.806 Da.

The antibody titer in rabbit plasma was determined by indirect enzyme-linked immunosorbent assay (ELISA) using an ELISA plate coated with peptide antigen of blapstin (6.06 mg/mL); the titer of purified polyclonal antibodies was more than 1:307,200, indicating that the peptide antigen had high accessibility. IgG was used as a positive control, and unimmunized rabbit serum was used as a negative control (see Fig. S2A in the supplemental material). The specificity of blapstin prepared using peptide as an antigen was verified by Western blot assay. The results showed a single band, indicating that the polyclonal antibody specifically recognized the blapstin protein (Fig. S2B). The result indicated that the polyclonal antibody of blapstin can be used to determine blapstin expression in different tissues. The relative expression of blapstin in different tissues of *B. rhynchopetera* was analyzed by ELISA and immunohistochemistry (IHC) experiments. These results indicated that the peptide was highly expressed in the fat body, followed by the hemolymph, midgut, and muscle, with the lowest expression in defensive glands (Fig. 3A and B). In addition, based on the standard indirect ELISA curve of blapstin (Fig. S2), it was determined that blapstin expression in the fat body and hemolymph was higher than that in the midgut, muscle, and defensive glands (Fig. 3C). These results indicated that blapstin may be synthesized in the fat body. Notably, as shown in Fig. 3D, the results of MALDI-TOF MS indicated the presence of blapstin in the midgut of *B. rhynchopetera* with an anisotopic molecular weight of 4,449.806 Da [(M + H)^+^, a mass discrepancy of (5 ppm)], which is a value equivalent to that of the renatured peptide of blapstin. Thus, blapstin is expressed in the midgut.

### Real-time PCR verified that selected fungi induce the expression of blapstin.

**FIG 4:**
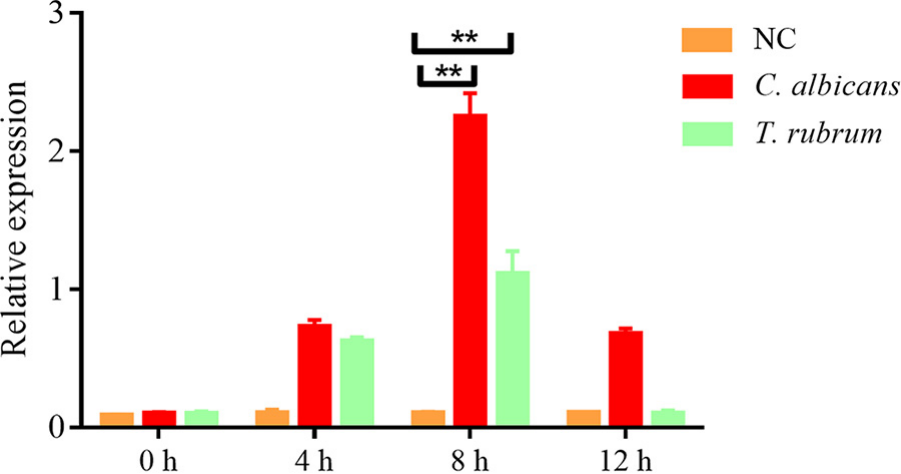
Real-time PCR verified that selected fungi induce the expression of blapstin. The negative control (NC) was represented by 0.9% NaCl. The results are expressed as the mean ± SD of three independent experiments. One-way ANOVA was performed; **, *P < *0.01.

The expression of blapstin was detected by real-time PCR after the fat body of RNA was extracted from the *B. rhychopetera* adults injected with C. albicans and T. rubrum (Fig. 4). The results showed that compared with the control group, the expression of blapstin was significantly upregulated when the *B. rhychopetera* adults were injected with the two strains, reaching the highest level at 8 h (Fig. 4; **, *P < *0.01) and then decreased at 12 h, which may be attributed to the mechanism that when *B. rhychopetera* was infected with C. albicans and T. rubrum, the expression of blapstin can be induced and the humoral immune response can be activated for defense (35).

### Antimicrobial activity of blapstin.

Blapstin did not affect bacteria, including Escherichia coli ATCC 8739 and Staphylococcus aureus ATCC 6538. However, it showed antifungal activity against Candida albicans ATCC 10231 and Trichophyton rubrum with MICs of 7 μM and 5.3 μM, respectively, indicating that blapstin has strong antifungal activity (Fig. S3; Table 1). In addition, three clinically isolated C. albicans strains, i.e., C. albicans 0065, 0063, and 6, were used in these experiments. The MICs of blapstin against the different strains of C. albicans ranged from 5.4 μM to 7 μM (Table 1).

**TABLE 1:** MICs are determined by blapstin acting on different microorganism strains

Microorganism and/or strain	MIC (μM)^*a*^		
	Blapstin	Colistin E	Fluconazole
Escherichia coli ATCC 8739	ND	3	ND
Staphylococcus aureus ATCC 6538	ND	81.1	ND
Candida albicans ATCC 10231	7	22	15
C. albicans 0065	3.5	5.4	70
C. albicans 0063	7	5.4	61
C. albicans 6	7	1.1	61
Trichophyton rubrum	5.3	ND	12.2

a ND, no bacteriostatic activity. Values are the mean results of three independent experiments.

In the tests of thermostability, pH stability, and the effect of proteases, blapstin was exposed to different temperatures (20 to 100°C) or pHs (3–9) prior to testing for antifungal activity toward C. albicans. From 20°C to 90°C, full antifungal activity was retained. The antifungal activity was slightly reduced when the temperature was elevated to 100°C (Fig. S4A and B). Thus, the antifungal peptide was thermostable up to 90°C. On the other hand, in the pH stability test, full antifungal activity was retained from pH 3 and pH 5. When the pH was higher than 7, the antifungal activity was reduced gradually (Fig. S4C and D). The results indicate that blapstin showed good activity in an acidic environment but decreased activity in an alkaline environment. The antifungal activity of blapstin was still significant after treatment with proteases for 1 h, including trypsin and chymotrypsin. The results revealed that blapstin was insensitive to trypsin and chymotrypsin (Fig. S4E).

**FIG 5:**
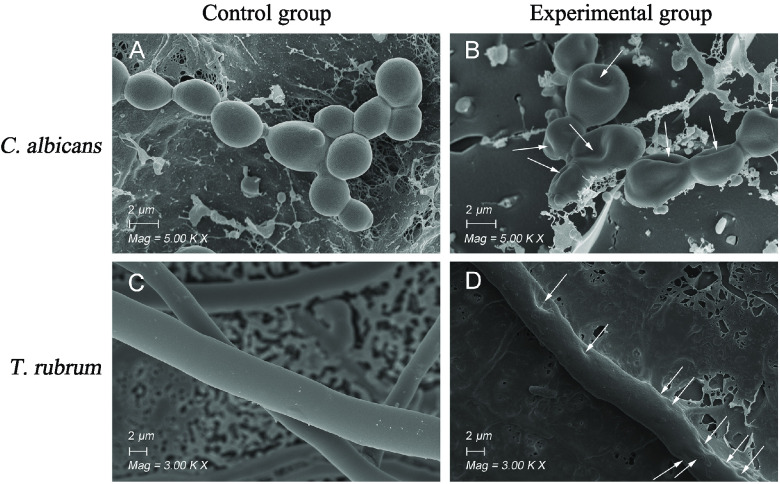
Blapstin kills fungi by affecting the fungal membrane. The fungi were treated with blapstin (1× MIC). Compared with that of a blank control (A and C), the membrane morphology of Candida albicans ATCC 10231 (B) and Trichophyton rubrum (D) under cryo-SEM became irregular and the thallus appeared concave. White arrows indicate the morphological changes in C. albicans ATCC 10231 and T. rubrum treated with blapstin. The blank control was represented by 0.9% NaCl.

To investigate the potential mechanism of blapstin acting on fungi, the morphological differences between untreated and treated fungal cells, including those of C. albicans ATCC 10231 and T. rubrum, were observed by cryo-scanning electron microscopy (cryo-SEM), and the membrane morphologies of treated and untreated C. albicans revealed a significant difference. The untreated C. albicans ATCC 10231 and T. rubrum showed intact cell walls and cytomembranes, and the adventitia was glossy (Fig. 5A and C). The C. albicans ATCC 10231 and T. rubrum treated with blapstin showed membrane shrinkage and disruption of the plasma membrane (Fig. 5B and D). These results suggest that the lesions might be directed to the plasmalemma of C. albicans ATCC 10231 and T. rubrum.

### Killing kinetics of blapstin against C. albicans.

**FIG 6:**
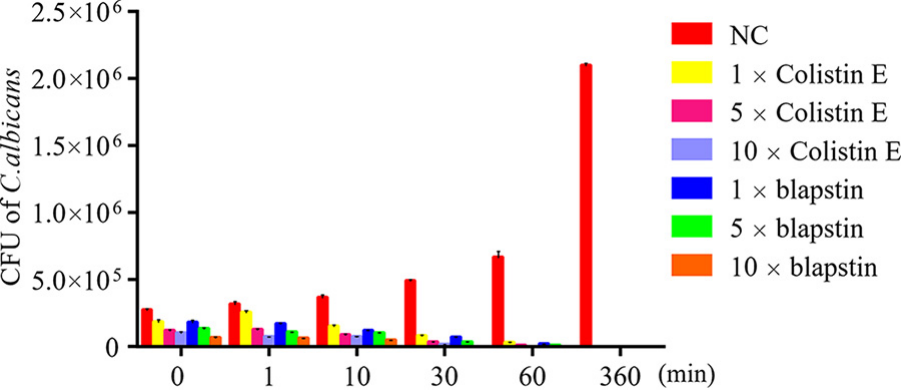
Killing kinetics of blapstin against C. albicans ATCC 10231. The negative control (NC) was represented by 0.9% NaCl. The results are expressed as the mean ± SD of three independent experiments.

Colony counting was performed to examine the anti-C. albicans effect of blapstin. With the positive-control drug colistin E, blapstin killed C. albicans ATCC 10231 in less than 60 min at concentrations of 5× and 10× MIC (Fig. 6). With an increase in incubation time and blapstin concentration, the effect of blapstin on C. albicans became stronger. The findings collectively demonstrate that blapstin exhibits efficacious antifungal activity.

### Hemolysis and cytotoxicity assays.

**FIG 7:**
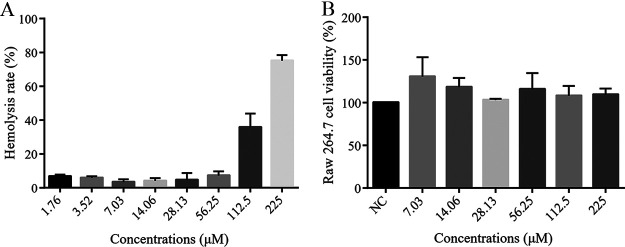
Hemolytic activity and cytotoxicity of blapstin. (A) Rate of hemolysis (%) of blapstin on human cells. Triton-100 was used as the positive control, and 0.9% saline was used as the negative control. (B) Cytotoxicity of blapstin on RAW 264.7 cells. NC, blank control containing sterile PBS without blapstin. The results are expressed as the mean ± SD of three independent experiments.

In contrast with the positive control Triton-100, blapstin showed little hemolytic activity against human erythrocytes even when the concentration was as high as 56.25 μM (Fig. 7A) and the HC_50_ value (peptide concentration causing 50% hemolysis of human erythrocytes) was more than 112.5 μM. Furthermore, no obvious cytotoxicity was observed in RAW 264.7 cells treated with different concentrations of blapstin (from 7.03 μM to 225 μM) (Fig. 6B). These results indicate that blapstin has low cytotoxicity.

### Effects of blapstin on biofilm.

**FIG 8:**
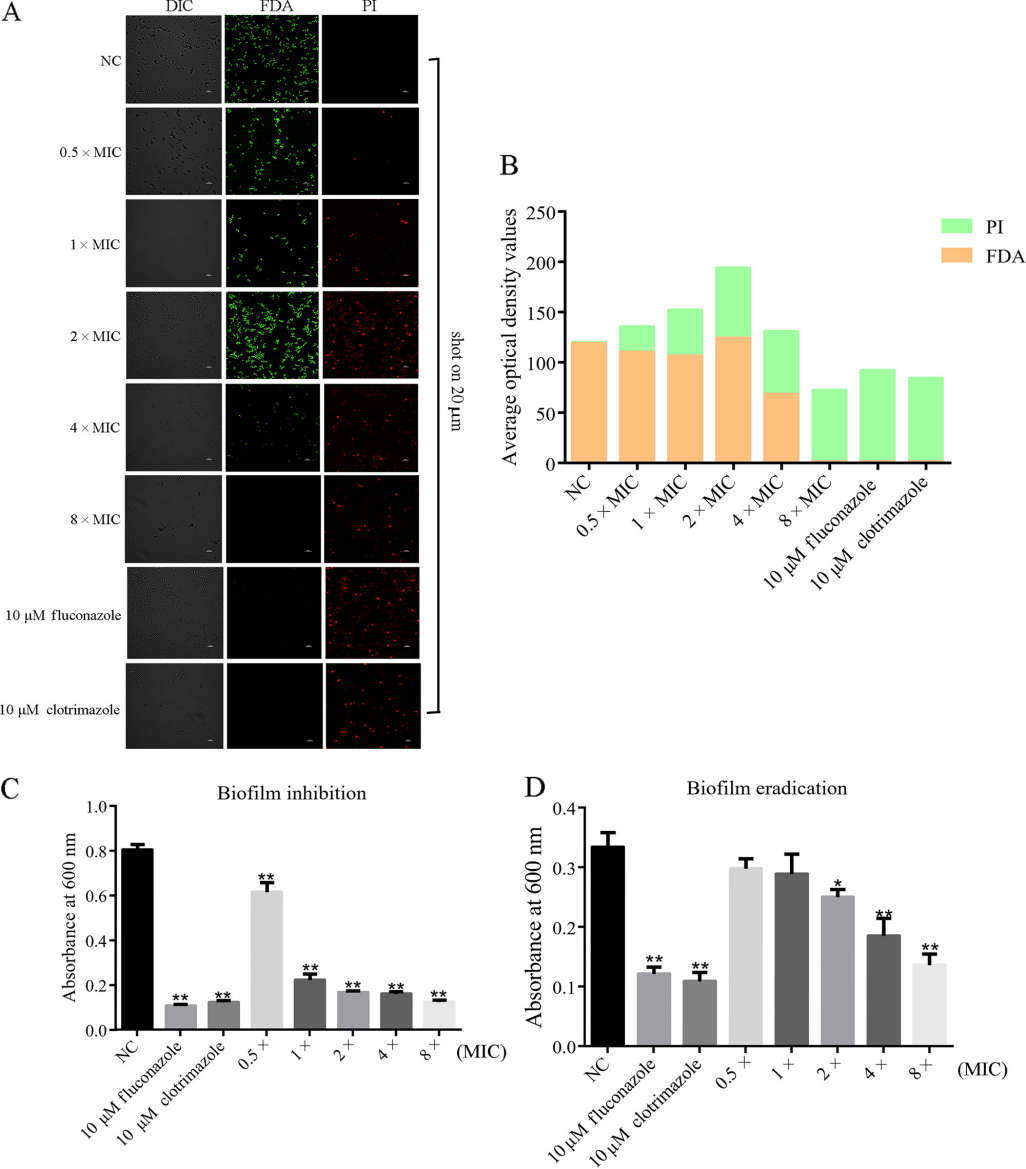
Effects of blapstin on biofilm formation. (A) Effect of blapstin on the biofilm of C. albicans ATCC 10231 by CLSM. Differential interference contrast was used to shoot in white light. Green fluorescence represents FDA-labeled cells, whereas red fluorescence represents PI-labeled cells. (B) Green or red fluorescence intensity was calculated using Image J. (C) Inhibitory effects of blapstin on biofilm formation of C. albicans ATCC 10231. (D) Effect of blapstin on eradication of established biofilms of C. albicans ATCC 10231. The negative control (NC) was represented by 0.9% NaCl. One-way ANOVA was performed; *, *P < *0.05; **, *P < *0.01.

Fungal biofilms are essential pathogenic factors and constitute a key feature leading to fungal infections (36). Therefore, we assessed the effects of blapstin on the biofilm-forming ability of C. albicans. The results indicated that blapstin dose-dependently inhibited biofilm formation by C. albicans (ATCC 10231) (Fig. 8A and B). In addition, blapstin also disrupted established biofilms of C. albicans in a dose-dependent manner (Fig. 8B). Moreover, the results from confocal laser scanning microscope (CLSM) showed that with increasing blapstin concentrations, the number and fluorescence intensity of fluorescein diacetate (FDA)-labeled live cells gradually decreased, whereas the number and fluorescence intensity of propidium iodide (PI)-labeled dead cells gradually increased. The 8× MIC of blapstin had the greatest effect on the biofilm activity of C. albicans ATCC 10231. Furthermore, under white light, the biofilm of blapstin-treated C. albicans ATCC 10231 appeared smaller and darker than that in control group (Fig. 8C). The findings indicate that the effect of blapstin on biofilm formation to a certain extent makes it a promising antibiofilm agent.

### Possible mechanism of blapstin on C. albicans biofilm.

**FIG 9:**
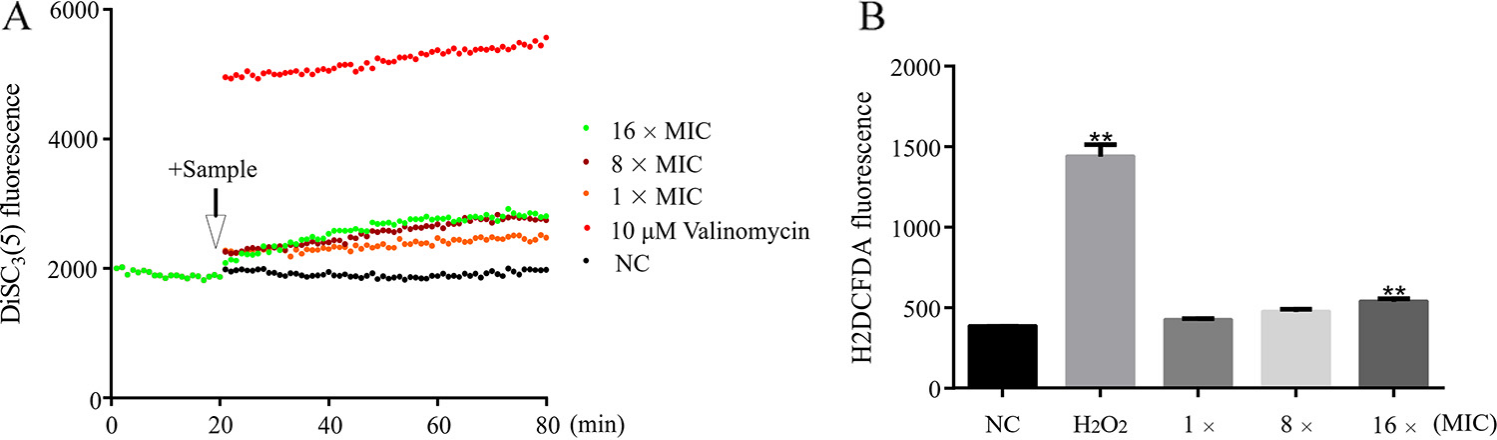
Possible mechanism of blapstin on biofilm of C. albicans. (A) DiSC_3_(5) staining monitors cell membrane potential changes. (B) H2DCFDA staining monitors the ROS burst. The negative control (NC) was represented by 0.9% NaCl. One-way ANOVA was performed; **, *P < *0.01.

In the test of effects of blapstin on the cell membrane of C. albicans ATCC 10231, treatment with 16× MIC, 8× MIC, and 1× MIC of blapstin resulted in an increase in the negative value of membrane potential and a change in the cells’ hyperpolarization. Cellular DiSC_3_(5) (3,3'-dipropylthiadicarbocyanine iodide) fluorescence increased significantly, compared with that of the negative control. Although blapstin did not have the same effect on the membrane potential changes of C. albicans as the positive-control valinomycin (Fig. 9A), the effect was in a dose-dependent manner.

The effect of blapstin on reactive oxygen species (ROS) burst was tested with a 2′,7′-dichlorofluorescin diacetate (H2DCFDA) probe. The results showed that the change in ROS fluorescence in C. albicans increased with the administration of blapstin, which indicated that blapstin could affect the change of ROS in C. albicans to some extent (Fig. 9B).

### Stability of blapstin in human plasma.

**FIG 10:**
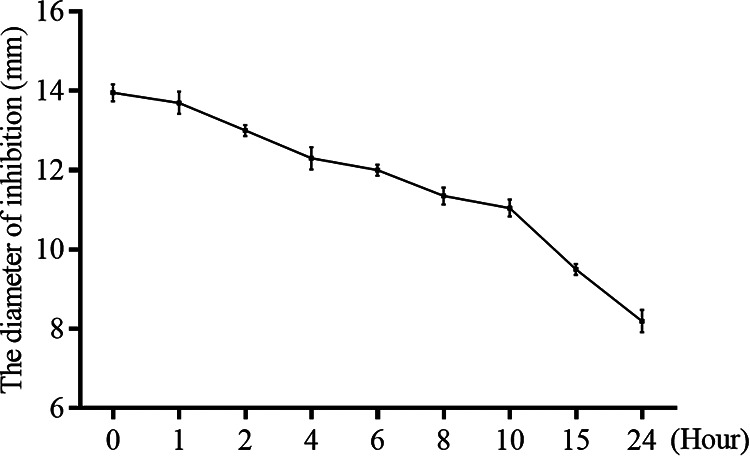
Stability of blapstin in human plasma. The antifungal activity of blapstin incubated with human plasma against C. albicans (ATCC 10231) for 0 to 24 h was assessed based on the diameter of the inhibition zone in a disk diffusion assay. The results are expressed as the mean ± SD of three independent experiments.

The stability of blapstin in human plasma was assessed based on the antifungal activity of blapstin. As shown in Fig. 10, the antifungal activity of blapstin decreased slowly with an increase in incubation time. Even after incubation for 24 h, the antifungal activity of C. albicans (ATCC 10231) did not completely disappear, indicating that plasma did not completely inhibit the antifungal activity of blapstin.

## DISCUSSION

AMPs are a class of small-molecule polypeptides that are induced and produced in insects and that are important effector molecules of insect innate immunity against microorganisms (37–40). Most AMPs isolated from insects have been reported to have antibacterial activity; however, some insects’ AMPs, such as DSP from *G. atrocyanea*, show antifungal activity (15, 41, 42). The present study describes the structure and characterization of a DSP-like peptide named blapstin from *B. rhynchopetera*.

Blapstin is a 41-amino-acid peptide; its sequence can be represented as CX_3_CX_9_CCX_10_CX_6_C, in which six cysteine residues form three disulfide bonds, similar to those in DSP (13). The six cysteine residues in ω-conotoxin GVIA form three disulfide bonds that have a compact and rigid conformation called an inhibitory cystine knot (ICK) motif (43–45). Therefore, from the structure of DSP, we speculate that the length between the second and third cysteine residues of blapstin is too long to meet the consensus sequence on the ICK motif and that the disulfide bond formation pattern of blapstin differs from that of ω-conotoxins and other antifungal peptides. Modeling studies indicate that the hydrophobic residues and adjacent basic residues on its β-sheet may allow a peptide’s structural components to interact with its targets (15); therefore, we speculated that the antifungal activity of blapstin is attributed to the interaction of these molecular structures. Nevertheless, unlike the DSP from the leaf beetle *G. atrocyanea*, blapstin did not show ion channel inhibition activity. This discrepancy may be related to the differences in their structure, which need to be further studied.

Insects, being invertebrates, cannot produce antibodies that specifically recognize invading microorganisms. Instead, they mainly rely on pattern recognition proteins (including AMPs) to initiate their defense responses to resist the invasion of foreign microorganisms (46). In this study, polyclonal antibodies were prepared to identify the expression of blapstin in different tissues of *B. rhynchopetera* adults. The results indicated that it was highly expressed in the fat body, followed by hemolymph, midgut, and muscle, with the lowest expression observed in the defensive glands. In addition, the reduction of 6.043 Da in molecular weight compared with the theoretical molecular weight of blapstin (4,455.853 Da) and an error of no more than 5 ppm suggest that blapstin is expressed in the fat body and hemolymph. Insect AMPs are a class of polypeptides produced by hemolymph and body tissues when insects experience microbial infection or receive accidental injury (47). Insect AMPs play an important role in humoral immune defense. The antimicrobial peptides synthesized by the fat body (similar to the liver of mammals) are secreted into the hemolymph to respond to systemic pathogens (48). The fat body is an important immune organ in insects and is the target secretory tissue of all major insect hormones, such as neurohormones, juvenile hormones, and ecdysone (49), and is also a reaction site for microbial infection. Hemolymph is distributed throughout all tissues, organs, and blood cells of insects and promotes the transport of nutrients, wastes, and metabolites. Thus, the high expression of blapstin in the fat body and hemolymph may represent a systemic immune response to an attack by foreign microorganisms. To a certain extent, blapstin expression in the fat body may reveal an innate immune function for *B. rhynchopetera*.

Fungal infections are not as common as bacterial infections, but are often more life threatening. Blapstin showed antifungal activity against C. albicans and T. rubrum; however, these two fungal species are not entomopathogenic. Certain fungal species that are harmful to humans cause diseases, including vaginitis and dermatitis. C. albicans may cause superficial mucosal infections in immunocompetent human hosts, among which vaginal candidiasis is the most commonly documented and encountered gynecological disease (50, 51). T. rubrum causes common superficial fungal skin diseases, such as psoriasis, tinea pedis, and tinea capitis (52, 53). In recent years, owing to the abuse of broad-spectrum antibiotics and immunosuppressants, the incidence of vaginitis and drug-resistant fungal species has increased annually. Vaginitis caused by drug-resistant C. albicans and skin infection caused by T. rubrum have become serious medical problems, and few specific agents have been found to completely cure the diseases. The types of antifungal drugs currently available are limited in comparison with antibacterial drugs, and the problem of drug resistance in fungi is more severe than that in bacteria mainly because fungi are eukaryotes and have more complex resistance mechanisms than bacteria. Therefore, identifying and developing antifungal drugs with natural active ingredients will help in the treatment of vaginitis and skin diseases.

Different insect antifungal peptides may have different mechanisms. The antifungal peptide polybia-MPI isolated from the venom of the social wasp Polybia paulista acts on C. albicans; polybia-MPI treatment leads to folded and cracked cell walls and inhibits the synthesis of mannan and chitin on the cell wall (54). Scolopendin 1 isolated from *Scolopendra subspinipes mutilans* adult centipedes is an antifungal peptide with a strong killing effect on C. albicans; it causes mitochondrial dysfunction, induces the ROS, and kills fungi by entering the fungal cells (55). The antifungal peptide papiliocin from the swallowtail butterfly Papilio xuthus acts on the fungal cell membrane to form pores and disrupt the cell membrane integrity (56). We speculated that blapstin acts on the cell membrane of C. albicans and T. rubrum, resulting in the appearance of depressions and holes and the leakage of fungal cell contents. Biofilm formation also signifies fungal growth to a certain extent (57). Blapstin exhibited marked biofilm formation inhibition and eradication activity against C. albicans strains in a concentration-dependent manner. These results are comparable with some previously reported findings which indicate that the antifungal activity of most insect antifungal peptides is achieved by damaging the lipid bilayer structure and targeting the fungal membrane (58). In addition, the low hemolysis, low cytotoxicity, and high plasma stability of blapstin add to the potential medical application of the peptide. Even at high concentrations, blapstin showed little hemolytic activity and cytotoxicity (Fig. 7). Notably, blapstin showed high stability in human plasma for 8 h at 37°C (Fig. 10). The selective antifungal activity, few side effects, and high stability make blapstin a potential candidate for the development of new antifungal agents.

## MATERIALS AND METHODS

### Insect and tissue collection.

*B. rhynchopetera* were collected from Yuanmou County, Chuxiong Autonomous Prefecture, in Yunnan Province, China. Adults were dissected to obtain a pair of defensive glands, midgut, and muscle, which were immediately preserved in RNAwait (Solaribo, Beijing, China) and stored at −80°C.

### cDNA synthesis.

Total RNA was isolated from *B. rhynchopetera* using TRIzol Agentia (Invitrogen, USA) according to the manufacturer’s instructions, and the purity of RNA was determined based on the *A*_260_/*A*_280_ ratio measured using a spectrophotometer (Maestro Nano, USA). The cDNA library was constructed using the SMART cDNA library construction kit (TaKaRa Bio, Japan) by following the manufacturer’s instructions, and the RNA was used as a template for reverse transcription (59). The first strand was synthesized using the SMART IV oligonucleotide (5′-AAGCAGTGGTATCAACGCAGAGTGGCCATTACGGCCGGG-3′) and the CDS III/3′ PCR primer [5′-ATTCTAGAGGCCGAGGCGGCCGACATG-d(T)_30_N_-1_N-3′]. The 5′ PCR primer (5′-AAGCAGTGGTATCAACGCAGAGT-3′) provided in the kit was used to synthesize the second strand using Advantage polymerase (TaKaRa Bio, USA).

### PCR amplification.

The cDNA samples were used as the templates in the PCRs: 3 min at 95°C for predegeneration and 35 cycles of 95°C for 30 s, 59°C for 30 s, and 72°C for 60 s, followed by incubation at 72°C for 5 min. The forward and reverse primers for blapstin were 5′-TCCAACACGGACTGCTTCG-3′ and 5′-CGAGGCGGCCGACATGTTTT-3′, respectively. The PCR products were analyzed using 1% agarose gel electrophoresis, and the gel segment containing the target band was sent to Sangon Biotech, Ltd. (Shanghai, China), for sequencing to obtain the complete sequence.

### Bioinformatics analysis.

The physicochemical properties of blapstin were predicted using the Expasy website (http://www.expasy.org/tools/). The transmembrane region and signal peptide region were analyzed through the TMHMM server, v.2.0 (http://www.cbs.dtu.dk/services/TMHMM/), and the SignalP 5.0 server (http://www.cbs.dtu.dk/services/SignalP/). CD was used to determine the secondary structure of blapstin, and the two-dimensional structure of blapstin was calculated by CD tool software (http://cdtools.cryst.bbk.ac.uk/). The three-dimensional structure of the peptide was predicted using the PHYRE2 online program (http://www.sbg.bio.ic.ac.uk/~phyre2/html/page.cgi?id=index) (60).

### Peptide synthesis.

The mature peptide of blapstin was synthesized by Sangon Biotech, Ltd. The purity was estimated to be >95% using reversed-phase high-performance liquid chromatography (RP-HPLC) and mass spectrometry (MS). The mature peptide of blapstin was dissolved in renaturation buffer (0.1 M Tris–HCl, 0.1 M NaCl, 5 mM glutathione [GSH], 0.5 mM glutathione oxidized [GSSG]; pH 5.86) and incubated at 26°C for 72 h (61). After the solution was filtered through a 0.22-mm filter, the peptide was purified by RP-HPLC (Waters, USA) on a C_18_ column (10 mm by 250 mm; Waters, USA). Solvent A was ultrapure water containing 0.1% trifluoroacetic acid (TFA), and solvent B was acetonitrile containing 0.1% TFA. The flow rate was 1.5 mL/min, and the detection wavelength was 280 nm (62). The column was balanced with 100% solvent A for 30 min and was eluted with 0% to 60% solvent B for 60 min. The refolded peptide was identified using MALDI-TOF MS (Autoflex; Bruker Daltonics, USA) and then lyophilized using a freeze dryer (Alpha 2-4 LDplus; Christ, Germany) and stored at −20°C.

### Preparation of polyclonal antibodies.

The protein antigen was mixed with an equal amount of Freund’s complete adjuvant (F-5881; Sigma, Darmstadt, Germany) during the first immunization and was fully emulsified. New Zealand White rabbits were immunized with subcutaneous multipoint injections for five stages to obtain polyclonal antibodies. At each subsequent stage, rabbits were injected with antigen mixed with Freund’s incomplete adjuvant (F-5506; Sigma, Darmstadt, Germany). Serum was collected after the fifth immunization. An indirect ELISA was used to detect the titer of polyclonal antibodies, and a Western blot assay was used to evaluate the polyclonal antibodies produced against the purified protein of blapstin; rabbit serum obtained before immunization was used as a blank control. The use of rabbits was approved by application for ethical approval for research involving animals of Kunming Institute of Zoology, Chinese Academy of Sciences (IACUC number IACUC-RE-2021-06-022).

### Expression of blapstin in different tissues of *B. rhynchopetera*.

The fat body, hemolymph, midgut, muscle, and defensive glands were collected from *B. rhynchopetera* in Yunnan Province. One portion was stored in 4% paraformaldehyde (pH 7.4) for IHC analysis. The other portion was placed in radioimmunoprecipitation (RIPA) buffer with 1% protease inhibitors and 1% phosphatase inhibitors, ground with a grinder (Servicebio, Wuhan, China), placed in an ice bath for 1 h, and then centrifuged at low temperature for 30 min (Eppendorf, Germany; model FA-45-24-11, 4°C, 1,000 × *g*). Protein concentrations of the supernatant of different tissues were determined using the bicinchoninic acid (BCA) method. The samples were diluted to the same level for ELISA analysis.

### ELISA analysis.

Each well of a 96-well ELISA microplate was coated with 100 μL of blapstin (the concentration in the first well was 0.45 μM, and serial double dilution was performed until the eighth well) to obtain a standard curve of blapstin (see Fig. S1 in the supplemental material). The supernatant of different tissue homogenates with the same protein concentration (final concentration, 10 mg/mL) was incubated with the coating buffer (0.015 mM Na_2_CO_3_, 0.035 mM NaHCO_3_; pH 9.6) overnight at 4°C; the plate was washed three times with Tris-buffered saline (TBS) including 0.05% Tween 20 (TBST). Blocking was performed by adding 100 mL blocking buffer (2% bovine serum albumin [BSA] in TBST) per well, and the plate was incubated for 40 min at 37°C. The plate was washed three times with TBST. Then, 100 mL of diluted rabbit anti-blapstin polyclonal antibodies (2% BSA in TBST; antibody titer, 1:307,200) (Fig. S2A) in blocking buffer was added to each well, and the plate was incubated for 1.5 h at 37°C. Unimmunized rabbit serum was used as the negative control. The KPL peroxidase-labeled antibody to rabbit IgG (H+L; Seracare, USA) diluted 1:4,000 with blocking buffer was then added to each well, and the plate was incubated in a shaking incubator for 40 min at 37°C. Next, the plate was washed four times to remove the unbound secondary antibodies, and 100 mL of tetramethylbenzidine (TMB) single-component substrate solution (Solarbio, Beijing, China) was added to the wells for 2 to 3 min at 25°C. Lastly, 100 μL of stop solution (Solarbio, Beijing, China) was added to each well to terminate the color development reaction, and the absorbance was read at 450 nm.

### IHC analysis.

After being fixed in 4% paraformaldehyde and dewaxing and dehydration, the tissue sections were washed in phosphate-buffered saline (PBS; pH 7.2). The sections were incubated in boiling citrate buffer (10 mM, pH 6.0) for 15 min to repair the antigenic site. Subsequently, the slices were incubated in 3% H_2_O_2_ for 25 min and washed three times in PBS (pH 7.4) on a horizontal shaker (Qilinbeier, Jiangsu, China) to block endogenous peroxidase activity and then blocked with 2% BSA at 37°C for 15 min. Afterward, the sections were incubated with purified rabbit polyclonal antibodies diluted at 1:1,000 at 4°C overnight and washed with PBS (pH 7.4). The sections were then incubated with the secondary antibody (KPL peroxidase-labeled antibody to rabbit IgG [H+L, Seracare, USA]) for 50 min at room temperature. The immunoperoxidase reaction was performed using 3,3-diaminobenzidine (Servicebio, Wuhan, China), and the sections were counterstained with hematoxylin. The primary antibody was replaced with an antigen-free antibody in the negative control. Images were captured using a slide scanning system (Teksqray, Shengzhen, China).

### Mass spectrometry analysis of blapstin in the midgut of *B. rhynchopetera*.

The midgut of *B. rhynchopetera* was dissected, flash-frozen in liquid nitrogen, and finely ground using a mortar and pestle. Then, the extraction solution (methanol-water, 1:1) was added and centrifuged at 5,000 × *g* for 10 min (Eppendorf, Germany; FA-45-24-11, 4°C). The supernatant was cracked by ultrasonic cavitation for 5 min, and then the solution was centrifuged at 5,000 × *g* for 10 min (Eppendorf, Germany; FA-45-24-11, 4°C), followed by ultrafiltration of the supernatant with a 10-kDa ultrafiltration tube. Based on the predicted molecular weight of blapstin (4,449.753 Da), the lower layer was analyzed using MALDI-TOF MS to verify the presence of blapstin in the midgut of *B. rhynchopetera*.

### Real-time PCR verifies that selected fungi induce the expression of blapstin.

C. albicans was cultured overnight and diluted in sterile YM liquid medium with 1.0 × 10^6^ CFU/mL, and T. rubrum was diluted in sterile Potato Dextrose Broth (PDB) with 2 × 10^5^ CFU/mL. The *B. rhychopetera* adults were injected with a volume of 2 μL per piece, 30 pieces in each group, and 0.9 NaCl was injected for the control group. Dissection took place at 0 h, 4 h, 8 h, and 12 h after injection. *B. rhychopetera* fat bodies were collected as anatomical objects and stored at −80°C. Total RNA was isolated using TRIzol Agentia (Invitrogen, USA), as described above in “cDNA synthesis.” RNA (200 ng) was a template for reverse transcription using oligo(dT) primer and 5× All-In-One MasterMix (with the AccuRT genomic DNA removal kit) (ABM, USA) in accordance with the manufacturer’s instructions. Samples of cDNA (1 μL) were used in real-time PCRs (constructed using the BlasTaq 2× qPCR mastermix kit [ABM, USA] in accordance with the manufacturer’s instructions) for 40 cycles of 95°C for 30 s, 59°C for 30 s, and 72°C for 60 s, followed by incubation at 72°C for 5 min. The forward and reverse primers for blapstin were 5′-TCCAACACGGACTGCTTCG-3′ and 5′-CGAGGCGGCCGACATGTTTT-3′, respectively. β-Actin (forward and reverse primers were 5′-GAAGTTGCTGCTCTGGTT-3′ and 5′-GGTGTGTTGAAGGTCTCG-3′, respectively) was the reference gene and was used as a control.

### Microorganism strains and growth conditions.

The standard strain of C. albicans (ATCC 10231), three clinically isolated antibiotic-resistant strains (C. albicans 0065, C. albicans 0063, and C. albicans 6), E. coli (ATCC 8739), and S. aureus (ATCC 6538) were obtained from Kunming Medical University. T. rubrum was purchased from BeNa Culture Collection (BNCC, CAS no. 340195). Different strains of C. albicans, E. coli, and S. aureus were cultured with shaking at 37°C in YM medium and Luria-Bertani (LB) medium. T. rubrum was cultured at 28°C in potato dextrose agar (PDA) medium according to the manufacturer’s instructions.

### Inhibition zone assay of blapstin.

Different strains of C. albicans and T. rubrum were inoculated on YM solid medium and PDA medium, followed by overnight incubation at 37°C and 28°C, respectively. For C. albicans strains (ATCC 10231, 0065, 0063, and 6), a single colony was picked with an inoculation loop, which was then used to coat a corresponding solid medium plate. Then, a sterilized 5-mm-diameter filter paper disk was placed on the surface of the medium, and 10 μL (225 μM) of blapstin was added to the filter paper, followed by incubation at 37°C for 18 h to observe the formation of an inhibition zone. The T. rubrum strain was inoculated on the PDA medium. When the colony grew to a diameter of 3 to 4 cm, 20 μL (225 μM) of blapstin was added around the fungal colony, and the plate was incubated at 28°C. Sterile 0.9% NaCl was used as the blank control. The antifungal effect of blapstin was assessed in comparison with that of the control group.

### Effects of temperature, pH, and protease on antifungal activity of blapstin.

**(i) Effects of temperature.** In the test of thermostability of antifungal activity, (20 μL（225 μM) of blapstin was heated at different temperatures (20°C, 40°C, 60°C, 80°C, 90°C, and 100°C) for 30 min and cooled down at 4°C (63). Colistin E was used as the positive control, and 1.5% NaCl was used as the negative control. Then, these samples were transferred to the paper disks in the YM solid medium with C. albicans and incubated at 37°C for 18 h for the assay of antifungal activity.

**(ii) Effects of pH.** In the test of pH stability of antifungal activity, 50 mg of blapstin was dissolved in 25 mL of solutions with different pH values (pH 3 to 5, 0.1 mol HCl; pH 7 to 9, 1 mmol NaOH), and the peptide solutions of different pH values were incubated at room temperature for 30 min (63). Colistin E was used as the positive control, and 1.5% NaCl was used as the negative control. Then, these samples were transferred to the paper disks in the YM solid medium with C. albicans and incubated at 37°C for 18 h for the assay of antifungal activity.

**(iii) Effects of protease.** In terms of the influence of protease on the antifungal activity of blapstin, trypsin and chymotrypsin were selected for the experiment, and 0.5 μL of a 10-mg/mL concentration of proteases (including trypsin and chymotrypsin) and (20 μL（225 μM) of blapstin were mixed and incubated at 37°C for 1 h (64). Colistin E was used as the positive control, and 1.5% NaCl was used as the negative control. Then, these samples were transferred to the paper disks in the YM solid medium with C. albicans and incubated at 37°C for 18 h for the assay of antifungal activity.

### Antimicrobial activity testing *in vitro*.

According to CLSI protocols, the MIC is determined as a point where there is “zero detectable growth,” The MIC of blapstin against different fungal strains was determined by use of the double dilution method, namely, broth dilution determination. In brief, the different strains were grown in their corresponding media at 28°C or 37°C until they reached the exponential growth phase. Colistin E and fluconazole were used as positive controls. Blapstin was diluted to various concentrations in sterile saline: 100 μL of sterile saline was added to the second well of a sterile 96-well plate, and then 100 μL of blapstin was added to the first and second wells. The solution from the second well was double-diluted with sterile saline until the 11th well, from which 100 μL was removed. In the 12th well, no blapstin was added, and sterile saline was used as a negative control. Afterward, 100 μL of the diluted microbial solution was added to each well, followed by incubation at 37°C for 16 h. To assess the antifungal activity of blapstin against the T. rubrum strain, the spore germination rates were first determined microscopically with a hemocytometer. T. rubrum was diluted with normal saline to 1 × 10^5^ CFU/mL and then incubated with various concentrations of blapstin at 28°C for 16 h. The absorbance at 600 nm was measured by a microplate reader (Epoch Etock; Biotek, USA) to estimate the inhibition of bacteria and fungi by blapstin.

### Cryo-SEM.

Cryo-SEM was used to examine membrane morphology as previously described with minor modifications (65). C. albicans ATCC 10231 was cultured in a nutrient YM solid medium. Sterilized 5-mm-diameter filter paper disks were placed on the surface of the medium, and 10 μL (1× MIC) of blapstin was added to the disks. Sterile 0.9% NaCl was used as the blank control. Fungi treated with blapstin were observed using cryo-SEM with a Quorum pp3010t system (Sigma 300; Carl Zeiss, Germany).

### Fungus-killing kinetics.

The *in vitro* killing curve for blapstin against C. albicans was determined as described previously with minor modifications (66). In brief, C. albicans ATCC 10231 was diluted to 2 × 10^5^ CFU/mL with sterile YM liquid broth. Different concentrations of blapstin (1×, 5×, and 10× MIC) were added to the fungal suspension and cultured at 37°C for various periods (0, 1, 10, 30, 60, and 180 min). Colistin E (1×, 5×, and 10× MIC) was used as a positive control, and 0.9% NaCl was used as a negative control. Within the specified time interval after incubation, the fungal suspensions were diluted 1,000-fold with YM medium, and 100 μL of the diluted fungal suspension was inoculated on YM solid medium. After incubation at 37°C for 24 h, colony counting was performed and the results were expressed as CFU per milliliter.

### Assays for hemolysis and cytotoxicity.

As previously reported (65, 67), hemolytic activity and cytotoxicity assays were performed using human erythrocytes and mouse macrophages (RAW 264.7 cells). These cells were obtained from Kunming Medical University and Cell Bank of Kunming Institute of Zoology, Chinese Academy of Sciences, respectively. To investigate the hemolytic activity, washed erythrocytes were diluted with normal saline into a suspension of 10^7^ to 10^8^ cells/mL and mixed with serial dilutions of blapstin (from 1.76 μM to 225 μM) at a 1:1 ratio, followed by incubation for 30 min at 37°C. Then, these erythrocytes were centrifuged for 15 min at 500 × *g* (Eppendorf, Germany; FA-45-24-11, 4°C). Next, the supernatant was collected, and the hemolysis rate (%) was measured to quantify the hemolysis of blapstin. The maximum hemolysis rate was determined using an equal volume of 1% Triton X-100 as a positive control, and the zero hemolysis rate was determined using sterile normal saline as a negative control. Hemolytic activity was calculated using the following formula: hemolysis rate (%) = (hemolysis of experimental group-negative control)/(hemolysis of positive control-negative control) × 100%. HC_50_ values were defined as the peptide concentrations causing 50% hemolysis of human erythrocytes.

Cytotoxicity was determined using mouse RAW 264.7 cells (10^6^ cells/mL). These cells were cultured in sterile 96-well microplates with RPMI 1640 medium (Corning, USA) in a 5% CO_2_ incubator at 37°C. The medium was supplemented with 10% fetal calf serum (FCS) and penicillin-streptomycin (100 U/mL each). Cytotoxicity was assessed using the cell counting kit-8 (CCK-8) assay (MCE, USA). After 24 h of incubation with serial dilutions of blapstin, 10 μL of CCK-8 solution was added to each well. The plate was further incubated for 1 to 4 h under standard cell growth conditions, and the residual absorbance was read at 450 nm. The experiments were repeated three times according to the manufacturer’s instructions.

### CLSM.

Blapstin at various concentrations (500 μL) was added to 500 μL of the prepared fungal suspension of C. albicans ATCC 10231 (2 × 10^5^ CFU/mL) in a sterile 24-well plate to ensure that the final concentration of blapstin ranged from 0.5× to 8× MIC; the plate was incubated for 48 h at 37°C, followed by washing with PBS two to three times to remove unadhered fungal cells. Then, 10 μg/mL of FDA (Solarbio, Beijing, China) and 5 μg/mL of PI (Solarbio, Beijing, China) were added for costaining in the dark for 30 min and washed with PBS twice, as described previously (68). Subsequently, the plates were observed using a CLSM (FV 1000; Olympus, Germany). The FDA and PI were excited at 488 nm and 555 nm, respectively, for imaging. Cell viability was assessed based on fluorescence staining; living cells hydrolyze FDA, resulting in the accumulation of green fluorescence, whereas dead cells are stained red by PI. Fluconazole at 10 μΜ and clotrimazole at 10 μΜ were used as a positive control, and sterile 0.9% NaCl was used as a negative control. Green or red fluorescence intensity was calculated using Image J.

### Inhibition of biofilm formation.

Biofilms are microbial aggregates produced by the extracellular matrix that are attached to abiotic and biotic surfaces (57). A biofilm inhibition assay was performed as previously described, with minor modifications, to determine the ability of blapstin to inhibit biofilm formation (69, 70). In brief, the concentration of C. albicans ATCC 10231 was adjusted to 1 × 10^6^ CFU/mL, and 200 μL per well was added to sterile 96-well plates mixed with different concentrations of blapstin (0.5×, 1×, 2×, 4×, and 8× MIC). After incubation for 24 h at 37°C, these wells were washed three times with PBS to remove unadhered fungal cells, followed by fixation with 99% methanol for 15 min. Next, methanol was removed, and the wells were allowed to dry. Then, 100 μL of 0.1% crystal violet was added to each well for 10 min, and the plate was washed three times. Finally, 100 μL of 95% ethanol was added, and the absorbance at 600 nm was measured to assess biofilm formation. Fluconazole at 10 μM and clotrimazole at 10 μΜ were used as a positive control, and sterile 0.9% NaCl was used as a negative control.

### Biofilm eradication assay.

First, 100 μL of C. albicans ATCC 10231 suspension (1 × 10^6^ CFU/mL) was added to each well of a sterile 96-well plate. The plate was incubated at 37°C for 24 h to make the biofilm adhere to the walls and washed three times with PBS. Next, 100 μL of serial dilutions of blapstin (0.5×, 1×, 2×, 4×, and 8× MIC) prepared using YM medium was added to the plate, followed by incubation at 37°C for 24 h. Fluconazole at 10 μM and clotrimazole at 10 μΜ were used as a positive control, and sterile 0.9% NaCl was used as a negative control. After incubation, the liquid in the plate was discarded and washed three times with PBS, followed by the addition of 1% crystal violet solution to each well and incubation at room temperature for 10 min. Finally, the plate was washed three times with PBS, and 100 μL of 95% ethanol was added per well. The percentage of biofilm removal was estimated by measuring the absorbance at 600 nm.

### Cell membrane potential changes.

DiSC_3_(5) is a fluorescent probe often used as a tracer dye to assess the mitochondrial membrane potential (71); therefore, we assessed the effects of blapstin on the cell membrane potential changes of C. albicans ATCC 10231 using DiSC_3_(5) staining. C. albicans ATCC 10231 was cultured in YM medium to logarithmic stage in a constant-temperature shaking machine at 37°C, centrifuged at 1,000 × *g* for 5 min to remove the supernatant, and washed with normal saline three times, and then normal saline containing 100 mM potassium chloride (KCl) was used to dilute the bacterial solution to an optical density at 600 nm (OD_600_) of 0.1. DiSC_3_(5) dye was dissolved by dimethyl sulfoxide (DMSO) into the mother solution and normal saline diluted with 15% DMSO so that the final concentration of DiSC_3_(5) added to the bacterial solution was 0.5 μM. The mixture was added to a special fluorescent 96-well plate with 190 μL in each well. The excitation/emission wavelengths were set at 622/670 nm, measurement was carried out with a multifunctional fluorescent enzyme labeling instrument (SynergyH1; Biotek, USA) until the value of the enzyme marker was stable, and then measurement was continued for 20 min. The final concentrations were 16× MIC, 8× MIC, and 1× MIC when 10 μL blapstin was added to the experimental group. In the positive-control group, 10 μL valinomycin was added and the final concentration was 10 μM. In the negative-control group, 10 μL 0.9% NaCl was added. Measurement of DiSC_3_(5) staining was continued for 60 min, and the experiment was repeated three times, with three biological replicates at a time.

### Detecting intracellular ROS burst.

H2DCFDA is a cell-permeable probe for detecting intracellular ROS (72). C. albicans ATCC 10231 was cultured in YM medium to logarithmic stage in a constant-temperature shaking machine at 37°C, centrifuged at 1,000 × *g* for 5 min to remove the supernatant, and washed with normal saline three times, and then the fungal solution was diluted to an OD_600_ of 0.1. Different concentrations of blapstin (1× MIC, 8× MIC, 32× MIC) were added to diluted fungal suspension and incubated at 37°C for 30 min. H_2_O_2_ at 0.1 mM was used as the positive control, and 0.9% NaCl was used as the negative control. After incubation, the suspension was centrifuged at 1,000 × *g* for 5 min to discard the supernatant and leave the thallus precipitation. The H2DCFDA dye was dissolved with DMSO and diluted with 0.9% NaCl containing 15% DMSO to achieve a final concentration of 10 μM. The thallus precipitation was mixed with 1 mL of a 100 μM H2DCFDA working solution to each tube, incubated at 37°C for 30 min, and then centrifuged at 1,000 × *g* for 5 min to remove the supernatant. The residual pellets were cleaned again with 0.9% NaCl to fully remove the H2DCFDA that did not enter the fungal pellets. One milliliter of 0.9% NaCl was added to each tube and analyzed with a multifunctional fluorescent enzyme labeling instrument (SynergyH1; Biotek, USA). The excitation/emission wavelengths were set at 626/670 nm, and the ROS burst was monitored. The experiment was repeated three times, with three biological replicates at a time.

### Effects of human plasma on the antimicrobial activity of blapstin.

The stability of blapstin in human plasma was assessed using a previously described method (67). Blapstin (225 μM) was mixed with 100% human plasma and incubated at 37°C for 0, 2, 4, 6, 8, 10, 15, and 24 h, respectively. Then, the residual antifungal activity against C. albicans ATCC 10231 for each incubation period was evaluated using an inhibition zone assay. Nutrient YM solid medium was coated with C. albicans ATCC 10231, and then 10-μL aliquots of the plasma-peptide mixture were added to sterile, 5-mm-diameter filter paper disks placed on the medium. After 24 h of incubation, the diameter of the inhibition zone against C. albicans ATCC 10231 was measured in triplicate.

### Statistical analysis.

All statistical data were analyzed using GraphPad Prism 6 (version 6.0.0; GraphPad Prism software). The results are expressed as the mean ± standard deviation (SD) of three individual experiments; one-way analysis of variance (ANOVA) was performed (*, *P < *0.05; **, *P < *0.01).

### Data availability.

The transcriptome data for the *B. rynchopetera* midgut was submitted to the National Center for Biotechnology Information (NCBI) using SRA accession number PRJNA935101, and the transcriptomic data set obtained from *B. rhynchopetera* defensive glands and muscle were submitted to NCBI under SRA accession number PRJNA767007. The peptide described here was named blapstin, and the sequence was submitted to GenBank under accession no. ON754988.
