# Supplementary material for: Blapstin, a Diapause-Specific Peptide-Like Peptide from the Chinese Medicinal Beetle Blaps rhynchopetera, Has Antifungal Function
Source: Microbiol Spectr. 2023 May 4;11(3):e03089-22. doi: 10.1128/spectrum.03089-22 (PMC10269622; doi:10.1128/spectrum.03089-22)
Supplement: Supplemental file 1 — Supplemental material. Download spectrum.03089-22-s0001.pdf, PDF file, 0.8 MB [file spectrum.03089-22-s0001.pdf]

Supplementary information

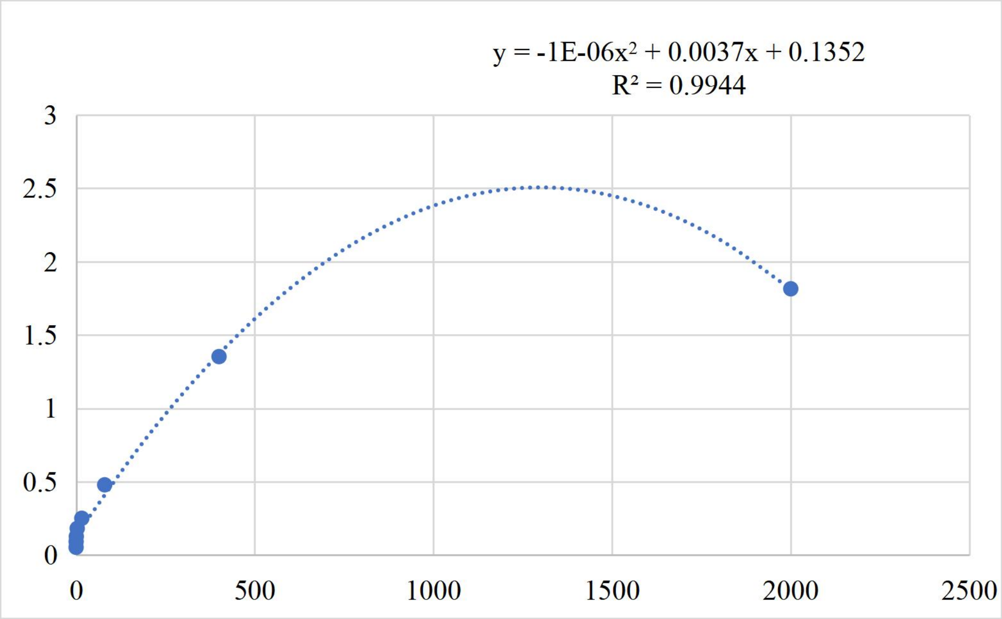

FIG S1 Blapstin standard curve drawing. The abscissa represented the concentration of blapstin (μg/mL), and the ordinate represented OD 450 nm of blapstin.

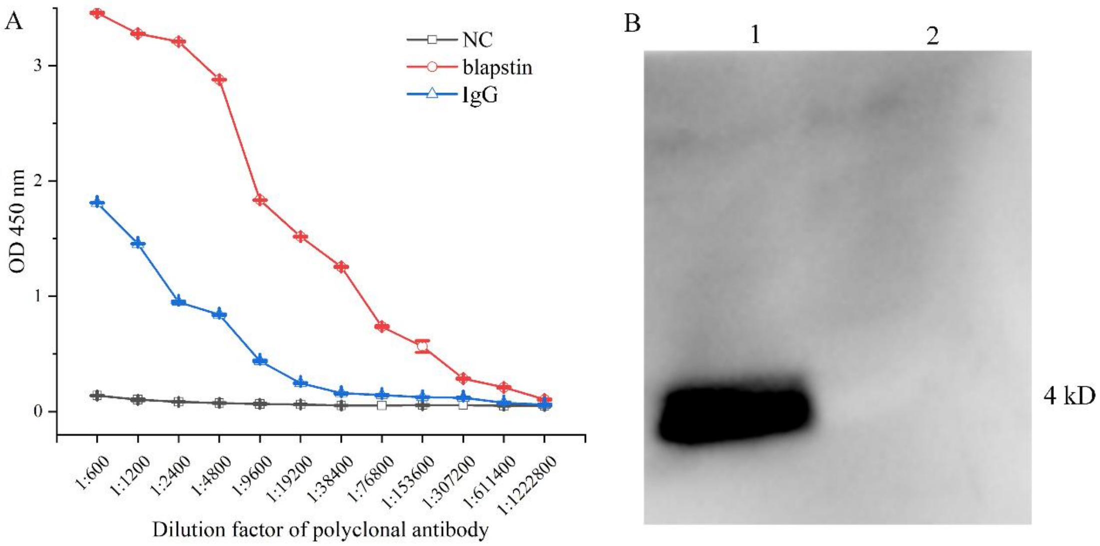

FIG S2 Preparation of polyclonal antibodies of blapstin. (A) Determination of the purified blapstin antibody titer (6.06mg/ml) by micro-plate reader at OD 450 nm based on indirect Elisa assay in the fifth immunization. (B) The specificity of the antibodies was verified by Western Blot analysis. (1, the polyclonal antibodies of blapstin, 2, serum without blapstin immune).

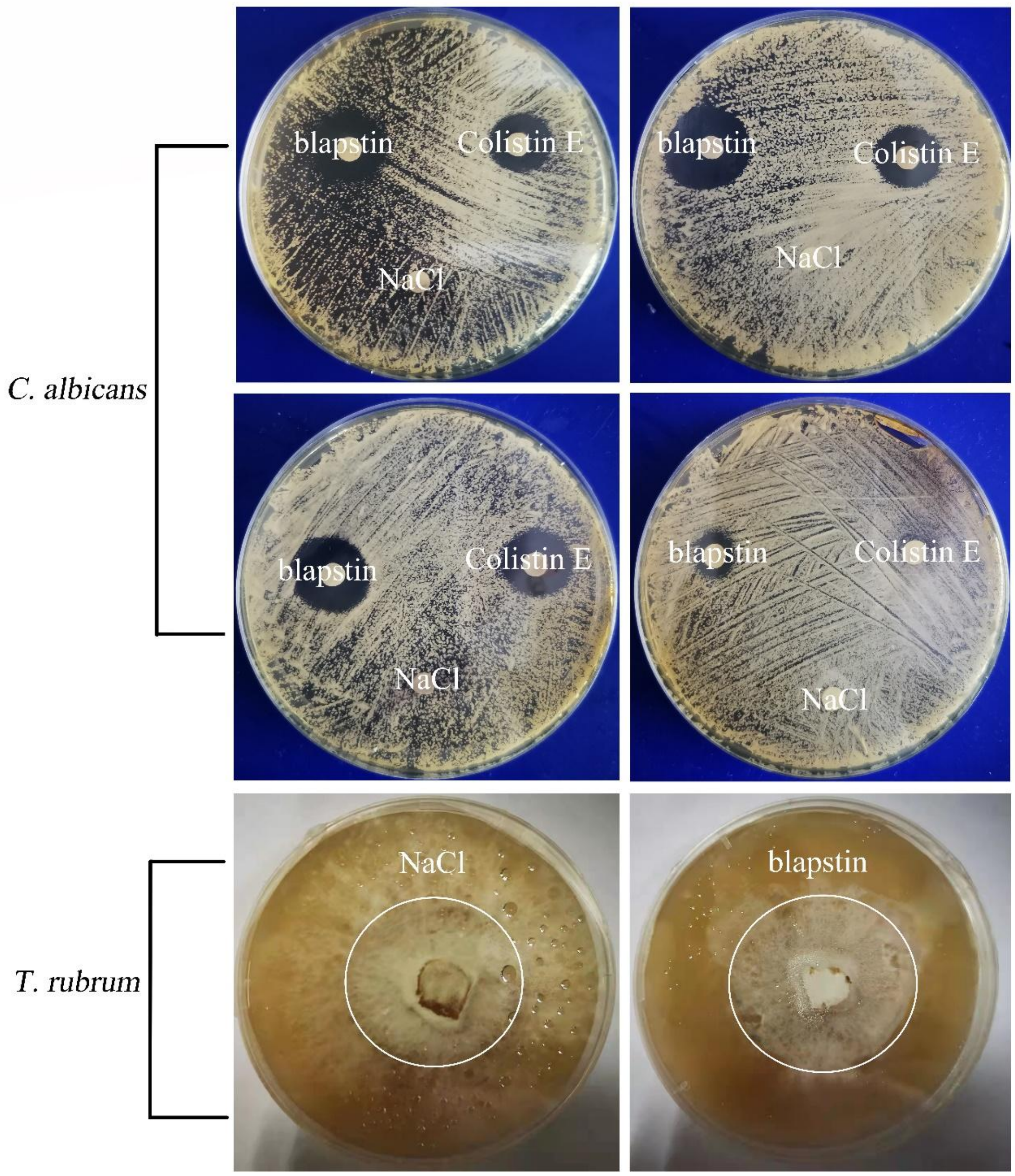

FIG S3 These strains of *C. albicans* and *T. rubrum* inhibition zone experiments on blapstin. The white circles represented the addition of blapstin and 0.9% saline around the *T. rubrum* when the diameter reached 3-4 cm to observe its bacteriostatic effect.

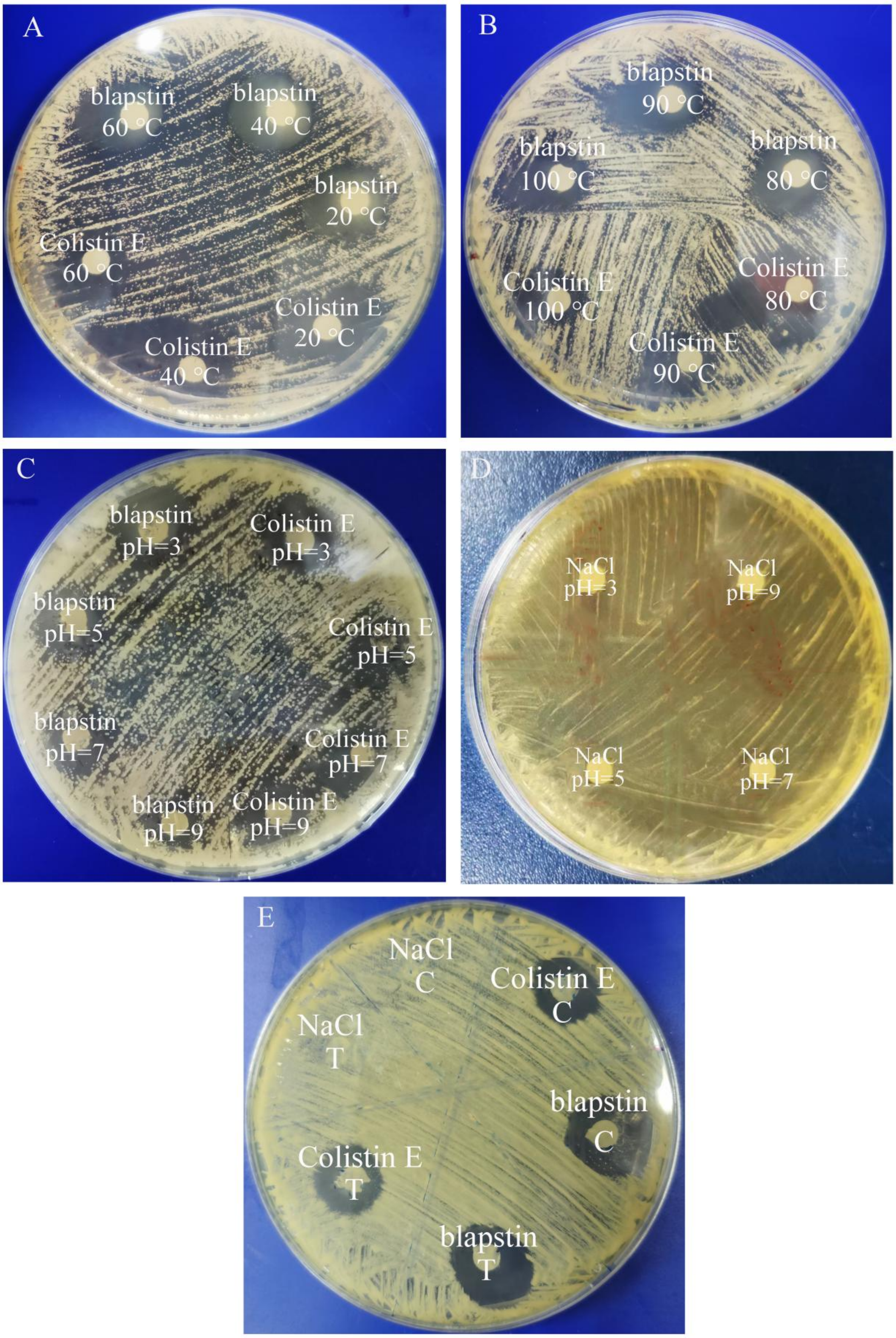

FIG S4 The effects of temperature, pH, and protease on antifungal activity of blapstin was performed to inhibition zone assay. A and B, the effects of temperature, including 20-100 °C. C and D, the effects of pH, including pH=3, 5, 7, 9. E, the effects of protease, including trypsin and chymotrypsin. ‘T’ was represented by trypsin and ‘C’ was represented by chymotrypsin.
